# Interaction Between Different Extracts of *Hypericum perforatum* L. from Serbia and Pentobarbital, Diazepam and Paracetamol

**DOI:** 10.3390/molecules19043869

**Published:** 2014-03-28

**Authors:** Aleksandar Rašković, Jelena Cvejić, Nebojša Stilinović, Svetlana Goločorbin-Kon, Saša Vukmirović, Neda Mimica-Dukić, Momir Mikov

**Affiliations:** 1 Department of Pharmacology, Toxicology and Clinical Pharmacology, Faculty of Medicine, University of Novi Sad, Hajduk Veljkova 3, Novi Sad 21000, Serbia; E-Mails: araskovic@hotmail.com (A.R.); sasavukmirovic99@gmail.com (S.V.); momir.mikov@otago.ac.nz (M.M.); 2 Laboratory for Pharmaceutical and Natural Products Analysis, Department of Pharmacy, Faculty of Medicine, University of Novi Sad, Hajduk Veljkova 3, Novi Sad 21000, Serbia; E-Mails: cvejich@hotmail.com (J.C.); magistrakon@orcon.net.nz (S.G.-K.); 3 Department of Chemistry, Biochemistry and Environmental Protection, Faculty of Sciences, University of Novi Sad, Trg Dositeja Obradovića 3, Novi Sad 21000, Serbia; E-Mail: neda.mimica-dukic@dh.uns.ac.rs

**Keywords:** naphtodianthrones, hypericin, pentobarbital induced sleeping time, rotarod, paracetamol plasma concentration

## Abstract

Herb-drug interactions are an important safety concern and this study was conducted regarding the interaction between the natural top-selling antidepressant remedy *Hypericum perforatum* (Hypericaceae) and conventional drugs. This study examined the influence of acute pretreatment with different extracts of *Hypericum perforatum* from Serbia on pentobarbital-induced sleeping time, impairment of motor coordination caused by diazepam and paracetamol pharmacokinetics in mice. Ethanolic extract, aqueous extract, infusion, tablet and capsule of *Hypericum perforatum* were used in this experiment. The profile of *Hypericum perforatum* extracts as well as paracetamol plasma concentration was determined using RP-HPLC analysis. By quantitative HPLC analysis of active principles, it has been proven that *Hypericum perforatum* ethanolic extract has the largest content of naphtodianthrones: hypericin (57.77 µg/mL) and pseudohypericin (155.38 µg/mL). Pretreatment with ethanolic extract of *Hypericum perforatum* potentiated the hypnotic effect of pentobarbital and impairment of motor coordination caused by diazepam to the greatest extent and also increased paracetamol plasma concentration in comparison to the control group. These results were in correlation with naphtodianthrone concentrations. The obtained results have shown a considerable influence of *Hypericum perforatum* on pentobarbital and diazepam pharmacodynamics and paracetamol pharmacokinetics.

## 1. Introduction

An increasing percentage of the population is using herbal products, and herbal products’ annual retail sales reflect this growing consumer interest. The reason for this wide usage is simple—people believe that being natural, all herbs are safe [1,2]. However, contrary to this perception, herbal drugs can cause serious adverse effects, as well as herb-drug interactions. In recent years, concerns about interactions between the natural antidepressant remedy St. John’s wort (SJW, *Hypericum perforatum* L., Hypericaceae) and conventional drugs have been raised [1,3].

St. John’s wort is an herbaceous perennial plant that is distributed worldwide. SJW extracts have become increasingly popular because of their reported beneficial effects on the nervous system [4,5,6]. SJW extracts contain at least 10 constituents or groups of components that could contribute to its pharmacological effects. These components include naphtodianthrones (hypericin and pseudohypericin), phloroglucinols (hyperforins), flavonoids and biflavonoids [7,8,9].

A potent central nervous system effect has been demonstrated, mainly for constituents belonging to the groups of naphthodianthrones (hypericin and pseudohypericin), phloroglucinols (hyperforin) and flavonol glycosides [1,3,10,11,12,13]. It is believed that they play major roles in the antidepressant-like effects of SJW. Today, in modern herbal medicine, SJW is used for mild depression more commonly than synthetic antidepressant medication. There are many commercial products based on SJW which are used and sold as dietary supplements. If SJW is applied simultaneously with some drugs, its active principles could show pharmacologically significant interactions with these drugs through induction of cytochrome enzymes and P-glycoprotein [1,3,14,15]. Beside these well-known interactions, there are also studies showing that hypericin, as one of the main components in SJW, can inhibit uridine 5'-diphosphoglucuronosyltransferases (UDP-glucuronosyltransferases), which catalyse the glucuronidation of a wide variety of xeno/endobiotics [14,16,17]. SJW and its active ingredients have been shown to inhibit the reuptake of several neurotransmitters such as serotonin, noradrenaline, dopamine, glutamate, and γ-aminobutyric acid which can lead to pharmacodynamic interactions with various xenobiotics [1,4,6,10].

Bearing in mind the widespread use of SJW and the consideration that herb-drug interactions are an important safety concern, the study was conducted in order to examine the interactions between different preparations of SJW from Serbia and pentobarbital, diazepam and paracetamol as examples of drugs that act on the central nervous system.

## 2. Results and Discussion

LC-MS analysis has been applied for a quick separation and identification of the major components of five different SJW preparations. Since there were no reference standards available for flavonoids and phloroglucinols, peak areas (divided by the injected mass of extract) were used as a measure of the absolute content of these classes. Four phloroglucinols (hyperfirin, adhyperfirin, hyperforin, adhyperforin) were identified together with several flavonoids (hyperoside, rutin, quercitrin, quercetin, biapigenin) and a small amount of caffeoylquinic acid (Table 1).

**Table 1:** Retention times and [M-H]^−^ ions of identified peaks.

Peak	*t_R_* [min]	[M-H]^−^, *m/z*	Compound
1	0.5	353	caffeoylquinic acid
2	0.7	609	rutin
3	0.77	463	hyperoside
4	0.94	447	quercitrin
5	1.72	301	quercetin
6	2.22	537	biapigenin
7	6.52	467	hyperfirin
8	6.68	481	adhyperfirin
9	7.08	535	hyperforin
10	7.18	549	adhyperforin

Peak identification was done by comparing the mass spectra and retention times with already published data [18,19]. The relative abundance of each group is determined by the normalization method, with the highest peak area being 100 for convenience. It is important to note that peak area, although useful for the comparison of the fractions’ purity, neither directly corresponds to the percentage composition in weight or amount, nor does it account for the total content of the compounds. Results obtained from the LC-MS analysis show that ethanolic extraction (ETE) gives the highest values for almost every compound extracted from SJW. On the contrary, water extraction yield (aqueous-AQE and infusion-INF) is much lesser than the ethanolic one for flavonoids (≈11 times) and phloroglucinols (≈72 times). There was almost no difference between ETE and AQE in the extraction of caffeoylquinic acid. This can be explained by the polarity of these compounds, with caffeoylquinic acid as highly polar, flavonoids as slightly polar and phloroglucinols as nonpolar. Highly polar molecules have similar solubility in water and ethanol, but most nonpolar molecules are water insoluble [11,12,13]. Furthermore, tablet (TBL) and capsule (CAP) has similar compositions, but still with lesser values than ETE (Table 2). According to these findings some of the further experiments were done.

**Figure 1:**
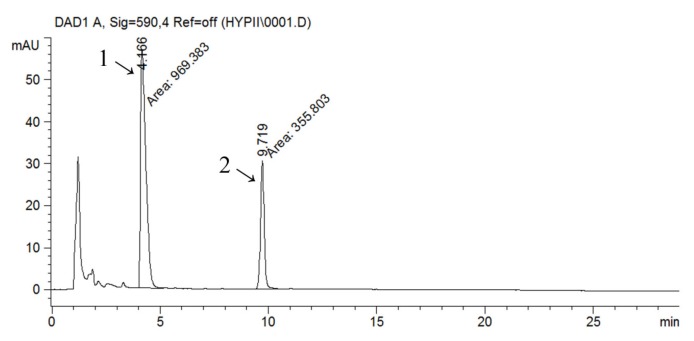
RP-HPLC chromatogram of ethanolic extract of *Hypericum perforatum* (ETE) detected at 590 nm; 1—pseudohypericin, 2—hypericin.

The HPLC-UV-DAD method was used for the identification and quantification of naphthodianthrones (hypericin and pseudohypericin). The chromatogram obtained by HPLC analysis of the ethanolic extract is shown in Figure 1 as a representative one. Other analysed extracts had similar chromatographic profiles. From a taxonomical and pharmacological standpoint, naphthodianthrones are marker components of *Hypericaceae* species [8]. Peaks 1 and 2 were identified as pseudohypericin and hypericin, respectively, by comparison with reference standards and literature data,. The peaks for hypericin and pseudohypericin were detected at 590 nm.

**Table 2:** Relative abundances * of identified compounds in different *Hypericum perforatum* extracts obtained from the LC-MS analysis.

Identified compounds	Extract				
	ETE	AQE	INF	TBL	CAP
caffeoylquinic acid	48	46	3	20	20
∑ phenolic acids	48	46	3	20	20
rutin	78	1.7	1.42	22.4	22.6
hyperoside	71	5.5	1.23	25.4	25.5
quercitrin	35	9.4	0.94	14.7	15.6
quercetin	100	10	0.13	94.8	96.9
biapigenin	6.9	0.04	0.10	11.9	11.9
∑ flavonoids	290	26.7	3.8	169.3	172.5
hyperfirin	40	0.18	0.36	20.8	16.6
adhyperfirin	25	0.12	0.18	28.3	26.5
hyperforin	8.3	0.70	0.17	3.6	3.7
adhyperforin	2.3	0.06	0.01	0.9	0.9
∑ phloroglucinols	76	1.05	0.71	54	48

* given as peak areas divided by injected mass, normalized to 100 for convenience.

Pseudohypericin is the main naphthodianthrone in SJW and is usually present in 2–5 times higher amounts than hypericin. A positive correlation was found between hypericin and pseudohypericin by various authors [7,8,9]. Quantitative HPLC analysis revealed that pseudohypericin concentration was higher than the hypericin’s in all of the analysed samples with a strong positive correlation between the concentrations of these two compounds. As for other investigated compounds, it has been proven that ETE has the highest content of hypericin (57.77 µg/mL) and pseudohypericin (155.47 µg/mL) and that water extraction gives lower concentration of these substances (Table 3).

Pentobarbital application at a dose of 40 mg/kg i.p. resulted in hypnosis in all animals tested, regardless of the pretreatment regime. The group of animals pretreated with TBL had significantly shorter sleeping induction time comparing to control group. Other investigated preparations also exhibit the same influence on duration of sleep induction time, but the effects were not statistically significant. All preparations except AQE prolonged sleeping time, increasing it up to more than 10 min. Pretreatment with ETE exhibited statistically significant effect on the CNS, by increasing sleeping time to 120 min (Table 4).

**Table 3:** Hypericin (HYP) and pseudohypericin (PHYP) concentration in different *Hypericum perforatum* extracts obtained from the HPLC-DAD-UV analysis.

Extract	HYP [µg/mL]	PHYP [µg/mL]
ETE	57.77	154.38
AQE	3.7	6.4
INF	4.35	18.54
TBL	20	77.45
CAP	14.24	53.69

**Table 4:** The effect of *Hypericum perforatum* extracts and preparations on pentobarbital-induced sleeping time (mean ± SD).

Group (*n* = 6)	Sleeping induction time [min]	Sleeping time [min]
Control	10 ± 4.9	60.8 ± 37.9
ETE	8.0 ± 2.9	120.7 ± 38.3 *
AQE	6.6 ± 5.2	57.7 ± 32.2
INF	7.7 ± 2.5	70.1 ± 48.4
TBL	4.3 ± 0.8 *	92.28 ± 12.9
CAP	7.2 ± 2.3	103.7 ± 25.5

* *p* < 0.05 compared with control group.

Results obtained in this study show that all examined SJW preparations administered four times in 24 h influence pentobarbital’s hypnotic effect, by shortening sleep induction and prolonging the duration of pentobarbital-induced sleeping time. Extracts used in this experiment show activity which is not in concordance with previously reported results of experiments performed with SJW extracts in the study of Jakovljevic *et al.*, who found that all used extracts of SJW shortened sleep induction and duration of pentobarbital-induced sleeping time. The shorter duration of pentobarbital-induced sleeping time in their study can be due to longer period of extract administration (12 days) which may have caused induction of pentobarbital metabolism [20]. The effect on duration of pentobarbital-induced sleeping time was most pronounced in animals treated with ETE. By quantitative HPLC analysis of active principles in these given products it was observed that ETE contained the highest concentrations of hypericin (57.77 µg/mL) and pseudohypericin (154.38 µg/mL) in comparison to other investigated preparations. As the sleeping time induced by pentobarbital is related to its central depressant properties, it could be modified by other factors such as pharmacokinetic interference. A short-duration acting barbiturate, pentobarbital is known to be almost completely hydroxylated by the cytochrome P-450 hepatic microsomal enzymes. Pentobarbital is metabolized by 3'-hydroxylation with hepatic microsomal enzymes, probably CYP2B6 and CYP2D6 [21]. In contrast to long-term SJW administration which leads to induction of cytochrome P-450 activity, there are reports showing that short-term administration can result in inhibition of the same enzymatic system [1,3,21], so the prolongation of sleeping time after pretreatment with different SJW preparations in our experiment could be due to inhibition of the metabolic pathway of pentobarbital in the liver.

The accelerating rotarod, where a rotating rod or drum functions as a treadmill for the rodent placed atop, is widely used to assess xenobiotic effects on motor coordination in rodents [22]. The effects of SJW preparations on forced-motor activity are presented in Table 5. The motor function impairment caused by diazepam was observed in all groups, except for CAP, in the 15th min (*p* < 0.05) in relation to control. In the 45th and the 60th min, ETE and INF impaired motor activity of mice, but the difference was not significant in comparison to control. In this experiment, as well as in the test performed with pentobarbital, ETE had the most pronounced effect.

**Table 5:** The influence of *Hypericum perforatum* preparations on motor coordination impairment in mice caused by diazepam (3 mg/kg, i.p.). The mean time of equilibrium maintenance is shown in seconds (mean ± SD).

Group (*n* = 6)	Time after diazepam administration			
	0'	15'	45'	60'
Control	300	234.17 ± 104	279.17 ± 51	300
ETE	300	30 ± 28.3 *	195 ± 144	197 ± 141.6
AQE	300	81.5 ± 91.6 *	280 ± 73.5	300
INF	300	67.83 ± 71 *	219.2 ± 128.8	230 ± 117.1
TBL	300	83 ± 45 *	282.5 ± 54.2	300
CAP	300	173.3 ± 139	300	300

* *p* < 0.05 compared with control group.

The results of this study confirmed the significant influence of SJW on the pharmacological effects of diazepam reported by Jakovljevic *et al.* [20]. Concerning this observation, in both studies the effect of the infusion was similar, and it lasted during all investigated times. In both studies, the effects of all analysed preparations were expressed more at the beginning of experiments (10 or 15 min after diazepam administration). The diazepam effect depends on its metabolism, where CYP-dependent hydroxylation and demethylation are the main processes. Diazepam is metabolised into the active metabolites desmethyldiazepam and oxazepam, which undergo glucuronidation to form glucoronide conjugates [23]. A possible explanation for these results could be that short-term treatment with SJW preparations inhibited CYP-dependent processes, as it was explained for pentobarbital tests [1,3]. Another explanation could be that SJW preparations inhibited glucuronidation of active diazepam metabolites and in that way prolonged and enhanced diazepam action on GABA receptors [16,17].

After calculation of the Pearson’s coefficient, there was an obvious correlation between concentrations of hypericin and pseudohypericin and the previously described parameters. The obtained results have shown a considerable influence of SJW on the hypnotic effect of pentobarbital and the impaired motor function of mice caused by diazepam. A strong positive correlation was observed for pentobarbital induced-sleeping time and both hypericin and pseudohypericin. Pretreament with SJW shortened equilibrium maintenance time, especially in the 15th minute after diazepam administration. These results show the significant influence of SJW on diazepam action which correlates with the hypericin derivative content (Table 6). Evidence available suggests that SJW and its active components have GABAergic activity [4,5]. Diana *et al.* proposed that hypericin may have an affinity for GABA receptors and that it could be one of the ways how it potentiates pentobarbital and diazepam effects. This is an example of pharmacodynamic interaction because pentobarbital and diazepam also act on GABA receptors [4].

**Table 6:** Prolongation of sleeping induction time (SIT), sleeping time (ST) and changes in equilibrium maintenance time at the 15th minute (t-15') in correlation with concentrations of hypericin and pseudohypericin (expressed in Pearson’s correlation coefficients).

Compound	SIT	ST	t-15'
conc. (hypericin)	0.25	0.87 **	−0.48 *
conc. (pseudohypericin)	0.12	0.92 **	−0.40 *

* correl > 0.4 ; ** correl > 0.8.

**Figure 2:**
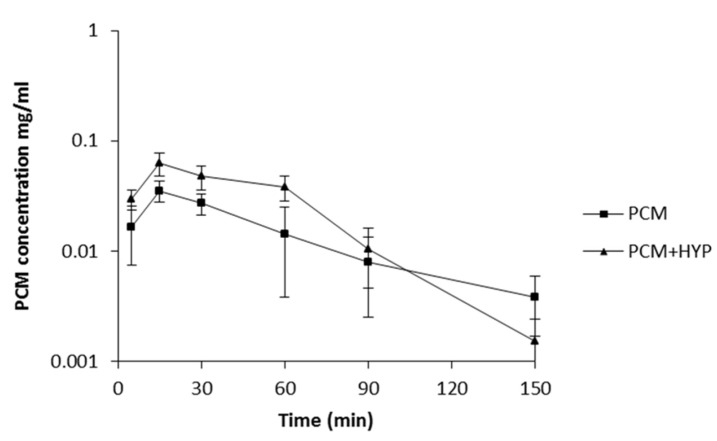
Paracetamol plasma concentrations in mice treated with paracetamol (200 mg/kg i.p.) only (PCM) and combination of paracetamol and ethanolic extract of *Hypericum perforatum* (PCM+HYP) (mean ± SD, *n* = 6), determined using HPLC-DAD-UV analysis.

As it was concluded from the previous tests that naphthodianthrone derivative content is important for interaction with pentobarbital and diazepam we decided to investigate the interaction between paracetamol and only ethanolic extract of SJW, which has the greatest concentration of hypericin and pseudohypericin. By comparison of the PCM and PCM + HYP experimental groups it was observed that there is a significant difference in paracetamol plasma concentrations. The PCM + HYP group had a significantly higher paracetamol plasma concentration in the 5th and 30th minute and one hour after administration of paracetamol than the PCM group. Paracetamol mean concentration *versus* time curves of the PCM and PCM + HYP groups showed that C_max_ of paracetamol is higher after SJW pretreatment (Figure 2).

The results of this study showed that pharmacokinetics of paracetamol could be altered by co-administration with SJW preparations rich in naphthodianthrone derivatives. Metabolism of the drug ocurrs primarily in the liver, via conjugation with glucuronic and sulphuric acids or cysteine. Glucuronidation accounts for up to two-thirds of paracetamol metabolism. A small amount of drug undergoes cytochrome P-450-mediated N-hydroxylation [24]. For the glucuronidation process the main responsible enzymes are UDP-glucuronosyltransferases. They require phosphorylation by protein kinase C for glucuronidation activity [17]. Volak and Court showed that this metabolic pathway could be compromised by protein kinase C-selective (rottlerin) and non-selective inhibitors (calphostin-C, curcumin and hypericin) [16]. In that study hypericin was presented as very potent protein kinase C inhibitor which could stop the activity of UDP-glucuronosyltransferases. That is one of the possible explanations for the statistically significant higher paracetamol AUC_(0-t)_ in the group of animals after short-term treatment with extract rich in hypericin. Although the AUC_(0-t)_ was significantly higher, parameters for evaluation of elimination processes, *i.e.*, K_e_, T_1/2_ and MRT, indicated that pretreatment with SJW induced paracetamol elimination (Table 7). As it was stated before, mean paracetamol plasma concentration in the 5th, 30th and 60th minute was higher in the PCM+HYP group but after that point it rapidly fell (Figure 2). SJW has been shown to induce P-glycoprotein and multidrug resistance-associated protein expression on the apical surface of the cells responsible for drug elimination. In addition, there are studies indicating that paracetamol could be a substrate for P-glycoprotein or multidrug resistance-associated proteins, thus induction of these efflux transporters could induce paracetamol elimination [25,26]. According to the results obtained in this pharmacokinetic study, it is obvious that short-term administration of ethanolic extract of SJW significantly altered the paracetamol metabolism and elimination process.

**Table 7:** Pharmacokinetic parameters of paracetamol in mice treated with paracetamol (200 mg/kg i.p.) only (PCM) and combination of paracetamol and ethanolic extract of *Hypericum perforatum* (PCM+HYP) (mean ± SD, *n* = 6).

Parameters	PCM	PCM+HYP
T_max_ (min)	15 ± 0	15 ± 0
C_max_ (mg/mL)	0.0353 ± 0.0075	0.0632 ± 0.0240
AUC_(0-t)_ (min·mg/mL)	2.06 ± 0.85	3.64 ± 0.96 *
K_e_ (min^−1^)	0.013 ± 0.005	0.0388 ± 0.013 **
T_1/2_ (min)	61.92 ± 24.93	19.11 ± 5.21 *
MRT (min)	72.93 ± 15.17	42.09 ± 5.54 **

* *p* < 0.05 *versus* PCM group;** *p* < 0.01 *versus* PCM group.

## 3. Experimental

### 3.1. Chemicals and Plant Material

Reference substances of hypericin (95%), pseudohypericin (95%) and paracetamol (99%) were purchased from Sigma-Aldrich (Steinheim, Germany). Pentobarbital sodium triethylammonium acetate and formic acid were also purchased from Sigma-Aldrich (Steinheim, Germany). Diazepam was obtained from Galenika (Belgrade, Serbia). Ultrapure water was generated with TKA water purification system, including reverse osmosis, activated carbon and ion-exchange cartridges. The acetonitrile and methanol used were of HPLC grade and obtained from J.T. Baker (Deventer, Netherlands).

Dried flowering tops of *Hypericum perforatum* L. were obtained from the Institute for Studies on Medicinal Plants, Josif Pančić, Belgrade, Serbia in 2008. Voucher specimen (*Hypericum perforatum* L. 1753 No. 2-1755, Serbia, grown in culture, 20 July 2008 det.: Goran Anackov) was confirmed and deposited at the Herbarium of the Department of Biology and Ecology, Faculty of Natural Sciences, University of Novi Sad (BUNS Herbarium, Novi Sad, Serbia).

Two marketed formulations were also obtained, tablet (TBL) and capsule (CAP). Tablet (TG-Farm d.o.o., Loznica, Serbia) used for these experiments contained 333 mg of SJW dry extract which is equivalent to 2000 mg of dried, powdered plant. One capsule (Quest Vitamins Ltd., Birmingham, UK) contained 100 mg of standardised SJW dry extract (0.368% hypericin).

### 3.2. Preparation of the Extracts

ETE: dry, powdered plant material (10 g) was macerated at room temperature for 72 h in 70% ethanol (150 mL). After filtration the solvent was removed under reduced pressure and the extract was dried and used.

AQE: dry, powdered plant material (10 g) was macerated at room temperature for 72 h in water (150 mL). After filtration the solvent was removed under reduced pressure and the extract was dried and used.

INF: dry, powdered plant material (3 g) was infused for 15 min in hot water (200 mL) and separated by filtration. The aqueous extract was concentrated under reduced pressure, dried and used in experiments as infusion. All dry preparations were dissolved in water to obtain the same concentration of 0.04 g of dry extract/mL.

*Marketed formulations*: 1 TBL and 1 CAP were dissolved in water in order to obtained solutions with concentration 0.04 g of dry extract/mL. Both preparations were used for experiments directly after filtration.

### 3.3. Animals and Extracts Administration

Experiments were carried out on adult sexually mature Swiss albino mice of both sexes, weighing 20–35 grams and of ages up to three months, which were bred at the Department of Pharmacology, Toxicology and Clinical Pharmacology at the Faculty of Medicine in Novi Sad, Serbia. Laboratory animals were under human care in accordance with the criteria given in the “Guide for the Care and Use of Laboratory Animals” edited by Commission of Life Sciences, National Research Council, USA. The study was approved by the Ethics Committee of the University of Novi Sad, (Novi Sad, Serbia) (No.: 01-153/6-2). Laboratory animals were quarantined and housed in Ehret UniProtect Air Flow Cabinet with High-Efficiency Particulate Air filter system (Emmendingen, Germany), at a controlled 21 ± 1 °C temperature and 55% ± 1.5% humidity with standard circadian rhythm (day/night). They had free access to a standard laboratory diet and water. The animals were randomly divided into test and control groups, each group consisting of six animals. Animals were treated with 0.04% water solution of each extract (400 mg/kg) orally, four times in 24 h (24, 18, 6 and 2 h before other tests). The control group received saline in the same volume.

### 3.4. Extracts Composition Identification by LC-MS

Chemical composition of fractions was determined by rapid resolution liquid chromatography with mass selective detection, using an Agilent Technologies 1200 Series liquid chromatograph coupled with an Agilent Technologies 6410B Series triple-quad (QQQ) mass spectrometer (Agilent Technologies, Santa Clara, CA, USA) using the method described by Orcic *et al.* [18]. Components were separated using reversed-phase Zorbax XDB-C18 50 mm × 4.6 mm, 1.8 μm column (Agilent Technologies), held at 50 °C. The mobile phase was delivered in gradient mode (0 min 25% B, 6 min 100% B, 8 min 100% B, solvent A being 0.1% aqueous formic acid, and solvent B being acetonitrile), with flow rate of 1 mL/min. Injection volume was 1 μL. Eluted components were ionized by electrospray ion source (ESI), using N2 for nebulization (pressure of 50 psi) and drying (flow of 10 L/min, temperature of 350 °C). Capillary voltage was 4000 V and fragmentor voltage 80 V. To increase the sensitivity, lower the noise, and simplify the spectra, negative ionization was used. Generated [M-H]^‒^ ions were analyzed using MS2SIM mode (MS1 experiment). For the purpose of LC-MS analysis, all dry extracts were dissolved in *iso*-propanol and for insoluble parts of each fraction, methanol was used.

### 3.5. Naphthodianthrones Identification and Quantification by HPLC-DAD-UV

Identification and quantification of naphthodianthrones was performed by HPLC analysis. The Agilent Technologies 1100 Series HPLC system consisted of a micro vacuum degasser, binary pump, Diode Array detector, autosampler, and Chem station software was used. All data processing as well as continuous on-line quantification and conditions control were performed by using Agilent Chem station software. Mobile phase component A consisted of a volume fraction of 1.0% triethylammonium acetate in water and component B consisted of acetonitrile. Gradient elution was used: From 25% B up to 100% B in 60 min. A 150 × 4.6 mm Zorbax SB-C18 (particle size 5 µm) column and a Zorbax SB-C18 (12.5 × 4.6 mm particle size 5 µm) guard column (Agilent) were used. The column was termostated at 25 °C, with a flow-rate of 1.0 mL/min. Injection volume was 10 µL and detection wavelength range was 220–750 nm. Analysis was carried out at the wavelength of 590 nm. All the analytes were quantified using peak heights.

### 3.6. Induced Sleeping Time Test

After pre-treatment with 0.04% water solutions of each extract animals received i.p. sodium pentobarbital at a dose of 40 mg/kg. Following pentobarbital injection, each mouse was observed for onset of sleep. A mouse reached criterion for sleep if it was placed on its back and exhibited a loss of righting reflex for 5 min. Mice that righted themselves in less than 5 min were considered to be awake. Sleeping induction time was recorded from the time of pentobarbital injection until 1 min after mice exhibited a loss of righting reflex. Sleeping time was recorded from 1 min after exhibiting a loss of righting reflex until regaining the righting reflex [27].

### 3.7. Rotarod (Forced Motor Activity) Test

The test for forced motor activity was conducted on animals that were able to maintain equilibrium on the fixed speed rotating rod for 5 min. Before the drug application, the mice were given one training trial during which they were allowed to remain on the rotarod up to 300 s. Two hours after last administration of water solutions of each extract, mice received i.p. diazepam at dose of 3 mg/kg. The selected animals were subjected to three consecutive trials on the rotating rod, 15, 45 and 60 min after administration of diazepam. In this test, coordination insufficiency produced by diazepam was indicated by the inability of the animals to maintain their equilibrium for at least 300 s on the rotating rod. Maintaining equilibrium was recorded for five minutes and mean time was expressed in seconds [27].

### 3.8. Measuring of Paracetamol Plasma Concentration and Other Pharmacokinetic Parameters

To investigate SJW extracts interaction with paracetamol we have measured paracetamol concentration in plasma of experimental animals. Two hours after the last dose of ethanolic extract of SJW, administered according to the previously described scheme, animals from experimental group paracetamol (PCM+HYP) received i.p. paracetamol at dose of 200 mg/kg (200 mg of pulverised paracetamol was dissolved in 10 mL of distilled water). Control for this group (PCM) received only paracetamol at above mentioned dose. Blood samples were collected from tail vein of animals with heparinised capillary tubes (20 µL volume) on 5th, 15th, 30th, 60th, 90th and 150th minute after administration of paracetamol. Obtained plasma samples were mixed with 40 µL of acetonitrile and extracted for 20 min at 40 °C in a thermomixer. Then samples were centrifuged for 10 min at 3000 g and supernatants were separated to a new tube and kept in a freezer at −20 °C until analysis.

Obtained samples were analysed using same HPLC-DAD-UV system as for quantification of hypericin and pseudohypericin in the extracts based on the method of Al-Obaidy *et al.* [28]. A 150 × 4.6 mm Zorbax SB-C18 (particle size 5 µm) column and a Zorbax SB-C18 (12.5 × 4.6 mm particle size 5 µm) guard column (Agilent) were used. Paracetamol UV detection was at wavelength of 254 nm after separation on the columns described above with isocratic elution using mobile phase (10% of acetonitrile and 90% of methanol). The column was termostated at 25 °C, with a flow rate of 1.0 mL/min and injection volume was 10 µL.

Paracetamol plasma concentration was quantified using peak height. Pharmacokinetic parameters of paracetamol, maximum concentration (C_max_), time to achieve C_max_ (T_max_), area under curve after extrapolation from time 0 to infinity (AUC_(0-t)_) elimination rate constant (K_e_), elimination half-life (T_1/2_) and mean residence time (MRT) were calculated using WinNonLin software, version 5.1, Pharsight (St. Louis, MO, USA).

### 3.9. Statistical Analysis

The level of significance between the groups was assessed with the Student’s t-test for small independent samples using MedCalc 9.2.0.1 software. Correlation was assessed with the Pearson’s product-moment correlation coefficient, using same software. All data are expressed as mean ± standard deviation (SD). A value of *p* < 0.05 was considered to be statistically significant.

## 4. Conclusions

In summary, we have demonstrated that the effects of the drugs tested can be modified through interference with their pharmacokinetic properties, especially metabolic pathways. We showed that hypnotic effect of pentobarbital, motor impairment caused by diazepam and paracetamol pharmacokinetic properties could be changed with short-term SJW co-administration. In addition, we have concluded that the content of naphthodianthrone derivatives, which can differ significantly due to extraction procedures, is important for the extent of the interaction.
